# Optimized sand tube irrigation combined with nitrogen application improves jujube yield as well as water and nitrogen use efficiencies in an arid desert region of Northwest China

**DOI:** 10.3389/fpls.2024.1351392

**Published:** 2024-05-24

**Authors:** Youshuai Bai, Hengjia Zhang, Shenghai Jia, Dongyuan Sun, Jinxia Zhang, Xia Zhao, Xiangyi Fang, Xiaofeng Wang, Chunjuan Xu, Rui Cao

**Affiliations:** ^1^ College of Agronomy and Agricultural Engineering, Liaocheng University, Liaocheng, China; ^2^ College of Water Conservancy and Hydropower Engineering, Gansu Agricultural University, Lanzhou, China; ^3^ Qinfeng Forestry Experimental Station of Minqin County, Wuwei, China

**Keywords:** in-suit infiltration test, jujube yield, nitrogen, sand tube irrigation, soil water storage, surface wetted area, WUE

## Abstract

Efficient water-saving irrigation techniques and appropriate nitrogen (N) application are keys to solving the problems of water scarcity and irrational fertilization in jujube cultivation. In this study, first, the effects of sand tube irrigation (STI) on surface and subsurface wetted characteristics were investigated using *in-situ* infiltration tests in a jujube garden. Compared with surface drip irrigation (SD), STI reduced surface wetted area by 57.4% and wetted perimeter of the surface wetted circle by 37.1% and increased subsurface maximum infiltration distance of wetting front by 64.9%. At the optimal sand tube depth of 20 cm, surface wetted area of the surface wetted circle decreased by 65.4% and maximum infiltration distance of the wetting front increased by 70.9%, compared with SD. Two-year field experiments then investigated the effects of STI and SD on soil water storage, jujube leaf chlorophyll, net photosynthetic rate, actual water consumption, fruit yield, and water (WUE) and N (NUE) use efficiencies at four levels of N (pure nitrogen: N1, 0; N2, 286 kg ha^–1^; N3, 381 kg ha^–1^; N4, 476 kg ha^–1^) at the same irrigation amount (45 mm irrigation^–1^, total of 8). Compared with SD, STI increased soil water storage 18.0% (2021) and 15.6% (2022) during the entire growth period and also chlorophyll content, nitrogen balance index, and net photosynthetic rate, with both increasing and then decreasing with increasing N. Compared with SD, STI increased yields by 39.1% and 36.5% and WUE by 44.3% and 39.7% in 2021 and 2022, respectively. Nitrogen use efficiency was 2.5 (2021) and 1.6 (2022) times higher with STI than with SD. STI combined with N3 had the highest yield, WUE, NUE, and net income and is thus recommended as the optimal water–N combination. In conclusion, STI combined with appropriate N application can be an effective water-saving irrigation technology alternative to SD in jujube cultivation in arid areas.

## 1 Introduction

Water scarcity is a major challenge that generally leads to the current unbalanced development in social and economic systems and ecosystem degradation in arid regions (Zhu et al., 2023). Based on World Meteorological Organization statistics on the state of the global climate in 2022, global mean temperatures have been the highest on record for the past eight years (WMO, 2023). This warming trend will increase evaporation and crop water requirements and therefore threaten the stability of crop production in arid areas (Yu et al., 2018; Habib-Ur-Rahman et al., 2022; Wang et al., 2022c). According to Food and Agriculture Organization statistics, between 691 and 783 million people faced hunger in 2022 (FAO, 2023). In addition, approximately 29.6% of the global population have unsustainable access to food, and about 900 million people suffered from serious food security problems. Thus, mankind is currently facing the multiple crises and challenges of water scarcity, climate change, and food security.

Micro-irrigation technology has provided strong impetus for sustainable development of agriculture in dry areas and has been widely applied in various crops (Madramootoo and Morrison, 2013; Mattar et al., 2021; Nazari et al., 2021; Zhang et al., 2022). Micro-irrigation delivers water and nutrients uniformly and accurately directly to the soil near crop roots at a relatively low flow rate through a pipeline system with emitters (Goyal, 2014; Liu et al., 2021). Drip irrigation is considered one of the most water-efficient micro-irrigation techniques. Integration of water and fertilizer technologies has significantly improved crop yields and water and nitrogen (N) use efficiencies and has been used extensively in arid agricultural production (Surendran et al., 2016; Fan et al., 2020; Ma et al., 2021; Cao et al., 2022). However, surface drip irrigation (SD) has a large, wetted surface area and also tends to form puddles in the saturated zone below the emitter, and therefore, surface evaporation cannot be ignored in arid environments. As a result, subsurface drip irrigation (SDI) is widely used to reduce surface evaporation and improve yields and water productivity (Payero et al., 2008; Kandelous and Šimůnek, 2010; Martínez and Reca, 2014; Sandhu and Irmak, 2022; Wang et al., 2022a). SDI reduces crop seasonal evapotranspiration by 39% because of water diffusion under the soil surface, compared with sprinkler irrigation (Valentín et al., 2020). Wang et al. (2022b) found that SDI increased yields by 19.8% compared with those with SD. SDI has gained momentum due to its advantages such as reduced evaporation and increased yields. However, drip emitter clogging is a major challenge in the application and development of drip irrigation technology (Niu et al., 2013). Similarly, drip emitter clogging is a primary obstacle to the widespread and effective application of subsurface drip irrigation technology (Niu et al., 2013; Muhammad et al., 2022). Muhammad et al. (2022) identified root invasion as the main cause of emitter clogging in subsurface drip irrigation systems. Therefore, it is urgent to explore new SDI technologies that can reduce surface evaporation, have higher flow rates and resist clogging.

Sand tube irrigation (STI) is a type of SDI in which a cylindrical soil pit is dug below the drip emitter and filled with fine sand (Meshkat et al., 1999, Meshkat et al, 2000). The fine sand can quickly infiltrate drip water down to a certain depth in the crop root system. In a laboratory experiment, Meshkat et al. (1999) reported that STI can reduce soil evaporation by about 26% over a 4-day period, compared with SD. In addition, a setup similar to that with STI for jujube trees improved jujube yield, fruit quality, and WUE and also significantly slowed secondary soil salinization (Sun et al., 2016). Bai et al. (2022) demonstrated that STI increased jujube yield and water use efficiency by increasing soil water storage and reducing soil water deficit. Therefore, STI may be one of the important alternative technologies to SD and conventional SDI.

To meet the quadrupling of global demand for food and feed, N fertilizer inputs in cropping systems increased fivefold from 1961 to 2015 (Zhang et al., 2021). Nitrogen is an essential ingredient in amino acids, proteins, nucleic acids, and chlorophyll and is therefore one of the most significant nutrient elements for plant growth and development and yield formation (Marschner, 2011; Larimi et al., 2014; Wen et al., 2020). Therefore, N utilization is one of the key factors in achieving high crop yields (Min et al., 2021). However, excessive N fertilization can lead to a series of negative effects, such as sharp declines in N use efficiency (NUE), increases in ammonia volatilization and greenhouse gas emissions, and increases in agricultural surface pollution (Zhong et al., 2016; Guo et al., 2021). Moreover, excessive N application always promotes crop vegetative growth, which inhibits the transformation of dry matter from vegetative to reproductive growth and thus reduces final crop yield (Wang et al., 2021c). Application of N fertilizers requires a compromise between increasing crop yields and avoiding serious environmental effects (Lassaletta et al., 2023). Additionally, effective improvement in NUE and reduction in ammonia volatilization are essential for sustainable agricultural development and environmental protection (He et al., 2022). Therefore, improvements in agricultural water productivity and NUE are important measures to ensure food security (Zhang et al., 2015; Kang et al., 2017).

Jujube (*Ziziphus jujuba Mill*.) has been cultivated for more than 4000 years in China. According to the 2021 China Rural Statistics Yearbook, the national production of jujube in 2020 and 2021 is 7.7 and 7.4 million tons, respectively (Department of Rural Socio-Economic Survey, 2021). It is cultivated over 3 million hectares and accounts for more than 98% of global production (Shen et al., 2021). This jujube fruit is popular among consumers for its flavor and high nutritional value (including vitamin C, amino acids, proteins and polysaccharides) (Rashwan et al., 2020; Dou et al., 2023). Jujube trees are ecologically and economically important tree species that are drought-resistant, can survive in barren lands, and play very important roles in higher incomes for farmers, environmental protection, and ecological construction in arid areas around the world. Currently, jujube sustainable production is constrained primarily by water shortages and increased production costs.

According to the above analyses, STI is more effective than SD in increasing soil water storage and reducing soil evaporation, while N is an extremely important nutritional element for crop physiology and growth. Therefore, it is necessary to investigate the effects of different N application rates on leaf chlorophyll, nitrogen balance index (NBI), photosynthetic rate,and water consumption of jujube trees under these two irrigation methods. Simultaneously, it is essential to investigate whether there is any room for further increasing yield and WUE through reasonable N application combined with sand tube irrigation. Although studies on subsurface drip irrigation have been conducted (Meshkat et al., 1998, Meshkat et al, 2000; Sun et al., 2016), few have conducted *in-situ* infiltration tests with STI or examined effects of jujube nitrogen regulation on soil water storage and jujube growth, yield, WUE, and NUE in arid desert areas.

Thus, the study aimed to: (1) determine the optimal STI technology parameters by exploring soil wetted characteristics of surface and subsurface vertical profiles; (2) investigate the effects of different N amounts under STI and SD on soil water storage, jujube leaf chlorophyll, NBI, net photosynthetic rate, actual water consumption, jujube fruit yield, WUE, NUE, and net income.

## 2 Materials and methods

### 2.1 Field *in-situ* soil water infiltration test

**Figure 1:**
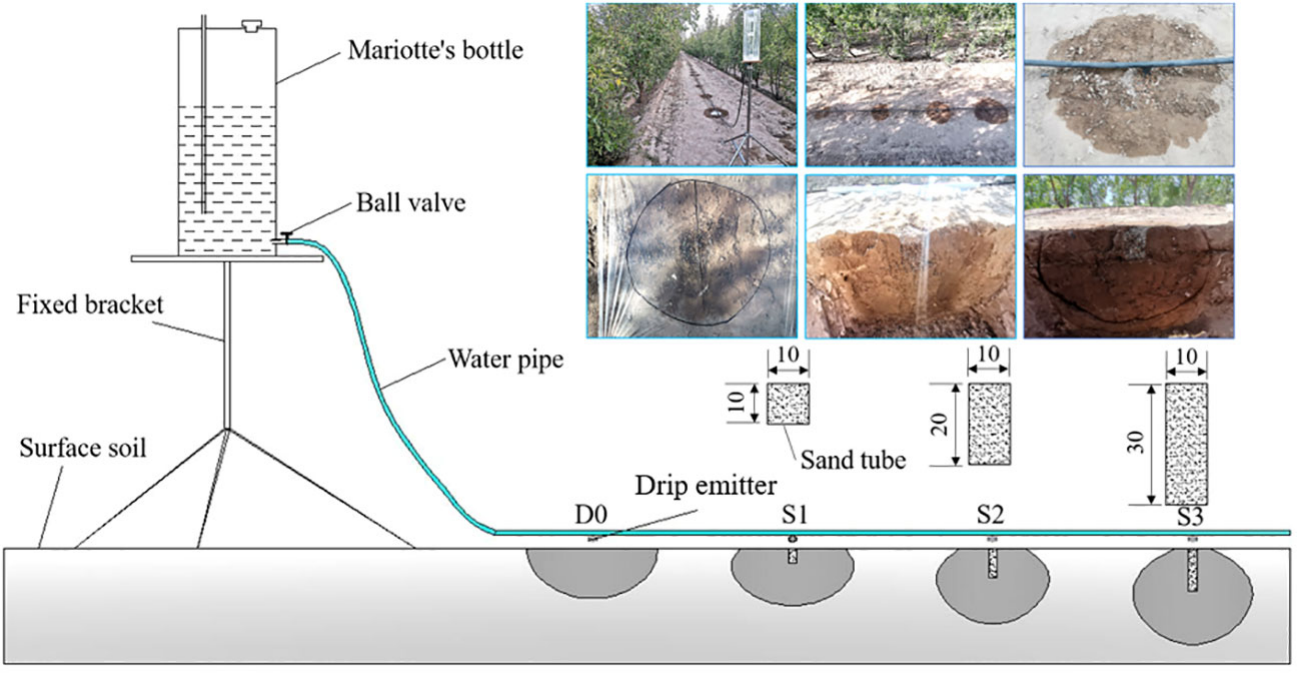
Schematic of the infiltration equipment.

The field *in-situ* soil water infiltration test was conducted in the jujube orchard (38°43’N, 103°01’E) of Qinfeng Forestry Experimental Station, Minqin County, Gansu Province, China. Following surface leveling, STI and drip irrigation infiltration systems were installed in dry soil of the jujube orchard on a sunny day. The soil moisture infiltration system consisted of a Mariotte’s bottle, a water supply pipe, drip emitters (flow rate = 4 L/h), a fixed bracket, and sand tube (filled with 2–3 mm in diameter of fine sand) laid below the drip emitter (**Figure 1**). The test was set up with four treatments: SD (D0) and STI with sand tube depths of 10 cm (S1), 20 cm (S2), and 30 cm (S3). There were three replications of each treatment. Sand tube diameter was 10 cm in STI treatments. All surface wetting was approximately circular because the irrigation was point-source drip irrigation. Thus, surface wetting was characterized by average diameter of a wetted circle, wetted area, and wetted perimeter. A ruler (mm) was used to measure the diameter of a wetted circle during the infiltration process. The wetted surface area and perimeter were determined based on the mulching film method (Guan et al., 2016). Specifically, at the end of the test, clean, clear film was placed on the wetted surface (including horizontal surface and vertical profile), and the location of the wetted front was drawn. Subsequently, a photograph was taken directly above the drawing, imported into Auto CAD 2014 software, and the wetting front on the picture was plotted with a spline curve. Then, the area and perimeter were determined with the AREA command. In addition, the vertical profile was located in the center plane of the sand tube (**Figure 1**).

### 2.2 Field experiment

#### 2.2.1 Experimental site and field conditions

**Figure 2:**
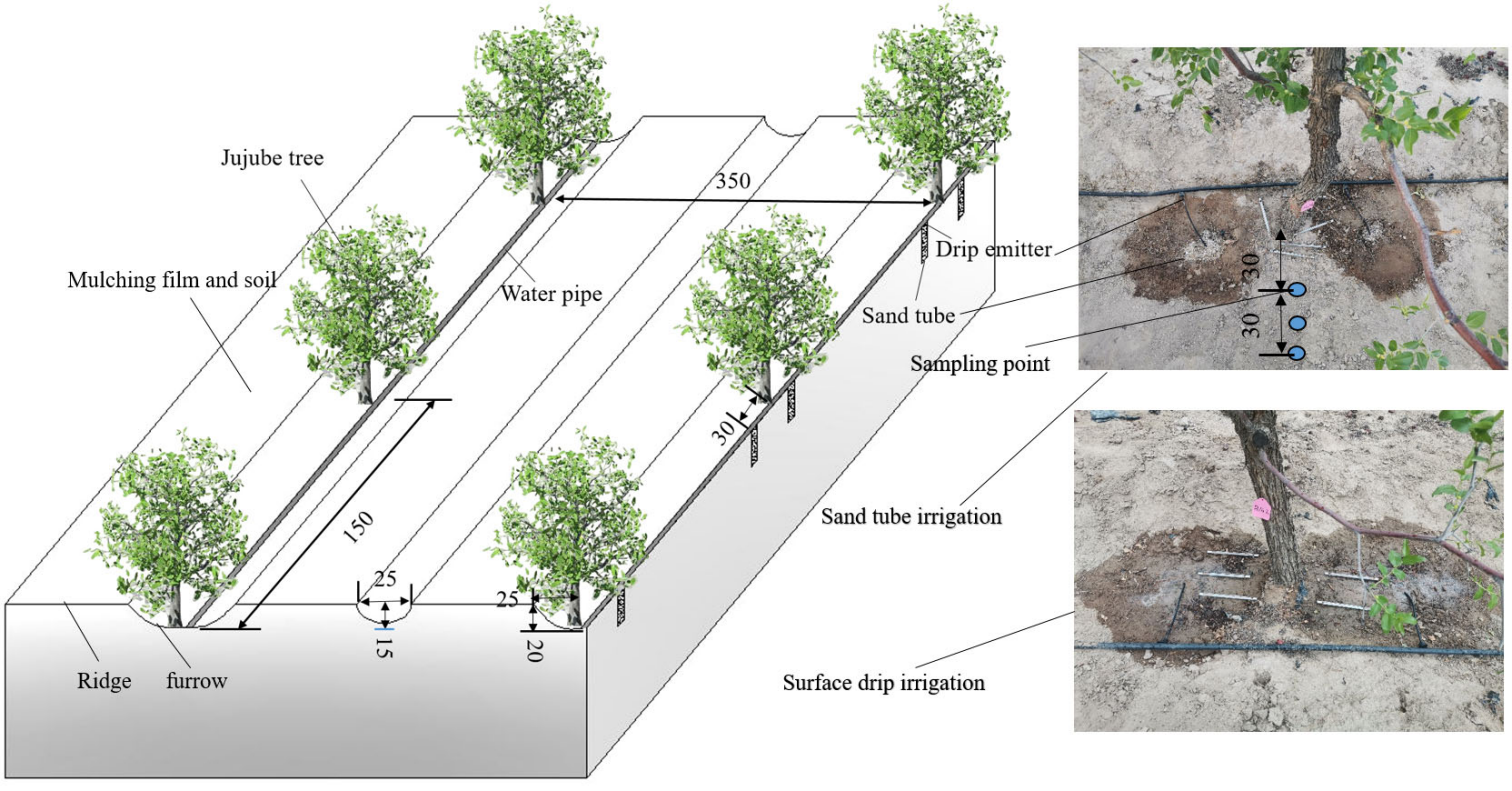
Design of field experiment.

The field experiments were conducted at the same locations as the *in-situ* soil infiltration tests (**Figure 2**). The climate of the area is continental desert climate. Annual average rainfall is 110 mm, and annual average air temperature is 7.8 °C. Groundwater depth is below 40 m. Average annual evaporation is 2,644 mm. Soil physical parameters are shown in **Table 1**. Field capacity and bulk density were determined by the cutting ring method (Duan et al., 2010), and soil particle size distribution was determined by the Laser Diffraction Particle Size Analyzer-Mastersizer 2000 (Malvern Instruments Co., Ltd, Malver, UK). According to the method of Bao (2000), soil nutrients contents were the following: organic matter: 5.3 g kg^–1^; total N: 0.4 g kg^–1^; total phosphorus: 0.5 g kg^–1^; total potassium: 17.1 g kg^–1^; alkaline dissolved N: 52.9 mg kg^–1^; effective phosphorus: 18.8 mg kg^–1^; quick-acting potassium: 85.1 mg kg^–1^.

**Table 1:** Soil physical parameters at different depths from 0 to 100 cm.

Soil depth (cm)	Soil particle size composition(mm)			Bulk density (g cm^−3^)	Field capacity (mass water content, %)
	<0.002	0.002–0.05	0.05–2		
0–20	14.87	48.77	36.36	1.43 ± 0.13	20.0
20–40	16.39	51.44	32.17	1.56 ± 0.07	
40–60	16.98	43.27	39.74	1.65 ± 0.06	
60–100	17.22	69.75	13.03	1.51 ± 0.15	16.17

#### 2.2.2 Field experimental design

Field irrigation experiments were carried out from May to October in 2021 and 2022 using 8-year-old jujube trees (*Ziziphus jujuba Mill, jun-jujube*) (**Figure 2**). The jujube trees monitored were of the same size (height of 250 to 300 cm), uniform and well-grown. Jujube tree row and plant spacing were 3.5 m and 1.5 m, respectively, and the field irrigation pipeline arrangement is shown in **Figure 2**. Irrigation water amount was controlled and monitored by a water meter (0.0001 m^3^). Irrigation water met the water quality (pH, Salinity, and Electrical conductivity of 7.2, 2.9 ppt, and 6.2 μS cm^–1^, respectively.) requirements for drip irrigation by filtration. Jujube trees were planted in furrows, and the rows were ridged with mulched film and a layer of soil.

To reduce surface evaporation and increase water content of deeper soils and preferential flow characteristics of plant root systems (Cui et al., 2021), a comprehensive assessment determined the optimal sand tube depth was 20 cm, with a tube diameter of 10 cm and with tubes filled with fine sand (2–3 mm in diameter). Each sand tube was arranged with a emitter with the 4 L h^–1^ flow rate. STI and SD were designed to provide the same irrigation amount (45 mm irrigation^–1^, total of 8) under four levels of N (pure nitrogen: N1, 0; N2, 286 kg ha^–1^; N3, 381 kg ha^–1^; N4, 476 kg ha^–1^) based on local, traditional fertilization experience. Nitrogen fertilizer type is urea (CO(NH_2_)_2_, 46.6% nitrogen). The method of fertilizer application is hole fertilization. Fertilizer application location is 30 cm away from the jujube tree, 20 cm in depth, and located on both sides of the jujube tree. The STI treatments were SN1, SN2, SN3, and SN4, and the SD treatments were DN1, DN2, DN3, and DN4. Each treatment was set up with 45 jujube trees as a plot and the area of each plot was 236 m^2^. Each treatment was three replications and three jujube trees were selected for each replication. Field irrigation and N application management strategies during different periods of jujube growth in 2021 and 2022 are shown in **Table 2**.

**Table 2:** Field irrigation and nitrogen application management strategies for different periods of jujube growth in 2021 and 2022.

Growth period		Leaf emergence	Flowering		Fruit swelling			Fruit maturation	
2021	Irrigation date	5.11	6.1	6.21	7.6	7.21	8.6	8.21	9.6
	Irrigation amount (mm)	45	45	45	45	45	45	45	45
	Nitrogen amount (total N%)	/	30%	30%	40%			/	
2022	Irrigation date	5.7	5.28	6.18	7.4	7.2	8.6	8.19	9.1
	Irrigation amount (mm)	45	45	45	45	45	45	45	45
	Nitrogen amount (total N%)	/	30%	30%	40%			/	

#### 2.2.3 Measurements and calculations

##### 2.2.3.1 Weather conditions

**Figure 3:**
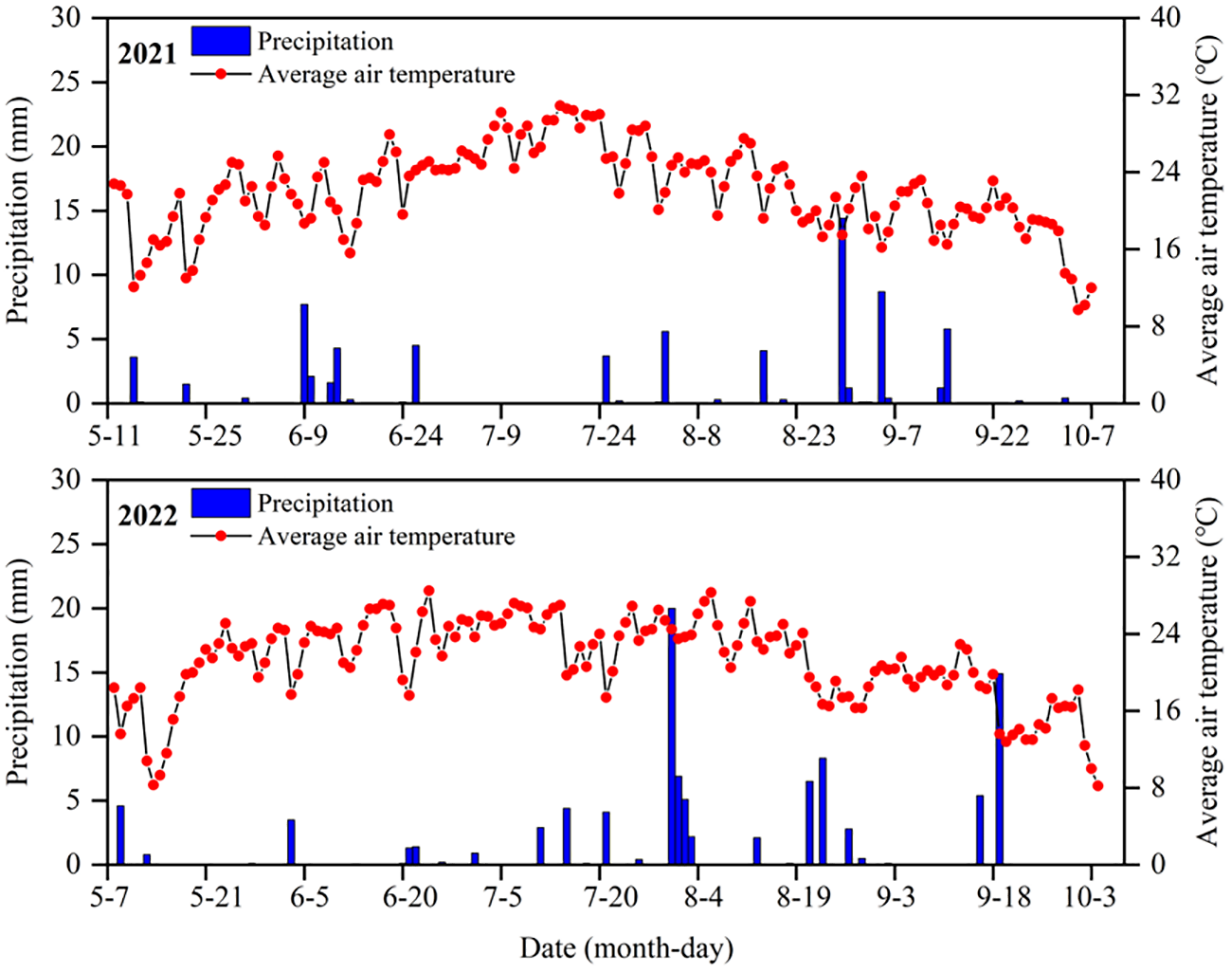
Daily precipitation and average air temperature during the growth period of jujube trees in 2021 and 2022.

Meteorological data of precipitation, relative air humidity, maximum temperature, minimum temperature, air pressure, solar radiation, and wind speed were recorded at 30-min intervals based on an automated meteorological monitoring station (TRM-ZS2, Sunshine Meteorology Co., LTD., Liaoning, China) installing at the experimental site. Precipitation was 82.5 mm in 2021 and 99.7 mm in 2022, of which the effective precipitation (≥5 mm) was 50.2 mm in 2021 and 67.1 mm in 2022 during the whole growth stages. In 2021, average monthly maximum temperature was 34.4°C in July, and average monthly minimum was 6.1°C in May. In 2022, average monthly maximum temperature was 33.2°C in July, and average monthly minimum temperature was 9.1°C in May (**Figure 3**).

##### 2.2.3.2 Soil water storage

Soil samples were collected at 30~60 cm (3 points uniformly) from the trunk of jujube trees with an iron soil auger (inner diameter of 40 cm) at 10-cm intervals from 0 to100 cm soil layer. Soil water content (gravimetric) was measured before and after each irrigation and determined by oven-drying. The original soil samples were re-backfilled after soil sampling. Each treatment was three replications. Soil water storage was calculated by the following formula (Liu et al., 2018):


(1) $$\;\;\;SWS=\sum_{i=10}^{n}H_{i}\;\times B_{i}\;\times \theta_{i}\;\times \;10^{-1}\;i=10,20,\ldots ,100\;$$

where *SWS*, *H_i_*, *B_i_*, and *θ_i_* are the soil water storage (mm), the soil depth (cm), the soil bulk density (g cm^–3^), and the soil water content (gravimetric, %), respectively, of the *i*th soil layer.

##### 2.2.3.3 Leaf chlorophyll content and net photosynthetic rate

Leaf chlorophyll content and NBI was measured by a Dualex 4 Scientific+ (FORCE-A, Orsay, France). Net photosynthetic rate (Pn) was measured with an Li-6400 photosynthesis system (Li-cor, Lincoln, USA). Net photosynthetic rate (μmol m^-2^ s^-1^) was measured at 10:00 AM on a clear morning. When measurements were taken, monitoring was repeated for three consecutive days and at one-week intervals. To minimize errors due to time points, the next monitoring was done in the reverse order of the previous one. Four leaves of each jujube tree with different orientations were measured, and the average value (replicated three times) was used as the final monitoring result.

##### 2.2.3.4 Yield, crop actual evapotranspiration, WUE, and NUE

Jujube fruits were harvested on October 7–8, 2021, and October 4–5, 2022. Jujube yield (Y, kg plant^–1^) was the all fruits weight of per tree.

Crop actual evapotranspiration (ET_a_, mm) was calculated by the following equation (Lu et al., 2021):


(2) $$\;\;\;\;\;\;\;ET_{a}=I+P+\Delta W-D-R\;\;\;\;\;\;\;$$

where I, P, ΔW, D, and R are the irrigation amount (mm), the effective precipitation, the soil water storage in the 0–100 cm soil layer from leaf emergence to fruit maturation periods, the deep seepage, and the rainfall runoff, respectively. Deep seepage and rainfall runoff were ignored because of the application of drip irrigation with low flow rates and no rainfall runoff during the entire jujube growth period.

Water use efficiency (WUE, kg m^−3^), and Irrigation water use efficiency (IWUE, kg m^−3^) were calculated by the following two formulas:


(3) $$\text{ WUE=}\frac{Y}{ET_{a}}\;\;\;\;\;\;\;\;\;\;\;$$

(4) $$\text{ IWUE=}\frac{\text{Y}}{I}\;\;\;\;\;\;\;\;\;\;\;\;$$

Nitrogen use efficiency (NUE, kg kg^−1^) was calculated by the following formula:


(5) $$\text{ NUE=}\frac{Y_{N}-Y_{0}}{M_{N}}\;\;\;\;\;\;\;\;\;\;\;$$

where Y_N_, Y_0_, M_N_ are the Y (kg ha^–1^) of an N fertilizer treatment, the Y (kg ha^–1^) of a no N fertilizer treatment, and the total N fertilizer amount (kg ha^–1^) for a treatment, respectively.

##### 2.2.3.5 Comprehensive analysis of economic benefits

The comprehensive analysis of economic benefits is calculated according to the following formula:


(6) $$E_{net}=E_{output}-E_{input}$$

Where *E_net_* is the net income ($ ha^−1^), *E_output_* is the total output ($ ha^−1^), and *E_input_* is the total input ($ ha^−1^). The total input includes irrigation water charge, fertilizer input, labor costs, and consumables such as irrigation pipes, emitters and their accessories. The irrigation water charge is based on the cost price of water published by the local government for that year. In addition, sand tube irrigation requires additional consumables (fine sand) and labor costs compared with surface drip irrigation.

### 2.3 Statistical analyses

Excel 2016 (Microsoft Corporation, WA, USA) was conducted to data analysis. OriginPro 2018 (Origin Lab, MA, USA) was used to create figures. With SPSS 22 software (SPSS Inc., Chicago, IL, USA), one-way ANOVA was used to assess treatment effects, Pearson’s correlation analysis was used to analyze the correlation between indicators under different treatments, and the LSD method was used to compare differences among means (*p* = 0.05). Auto CAD 2014 software (Autodesk, CA, USA) was used to create partial sketches and perform calculations of wetted soil area and perimeter.

## 3 Results

### 3.1 Soil water transportation under STI and SD

#### 3.1.1 Diameter of surface wetted circle

**Figure 4:**
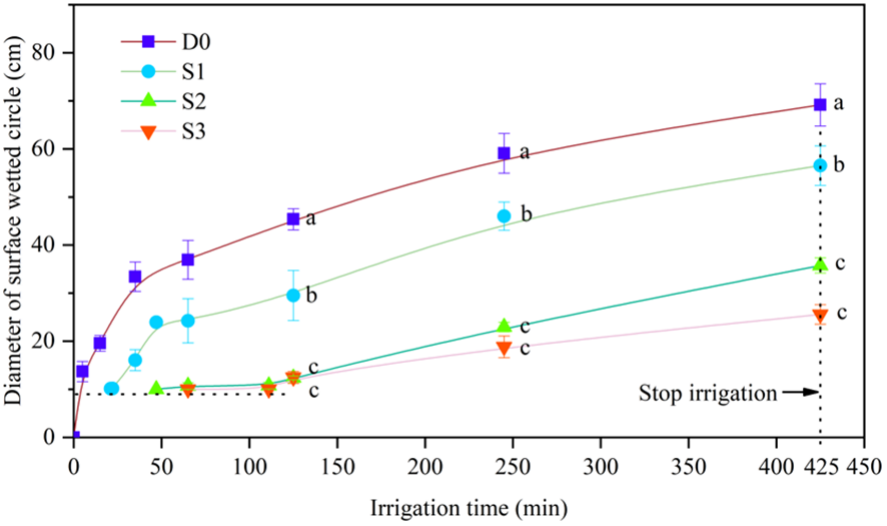
Variations in diameter of surface wetted circle with irrigation time. Different lowercase letters indicate significant differences at p< 0.05. Bars indicate the standard error.

The relation between diameter of surface wetted circle (D_c_) and irrigation time is shown in **Figure 4**. The D_c_ increased with increasing irrigation time in each treatment. During the entire infiltration process, SD (D0) diameter was significantly (*p*< 0.01) larger than that with STI, although surface wetting occurred in S3. At the end of irrigation, the D_c_ of D0 was 1.2, 1.9, and 2.70 times larger than that in S1, S2, and S3, respectively. In addition, the D_c_ in S1 was significantly larger than that in S2 and S3 by 58.1% and 121.0%, respectively, whereas there was no significant difference between S2 and S3. During the experiment, a surface water puddle was discovered at 15 min of irrigation in D0 and at 115 to 121 min in S1. Surface water puddles did not form in either S2 or S3 when irrigation ceased. At the beginning of infiltration, surface wetting in S1, S2, and S3 first appeared at 21 min, 47 min, and 111 min, respectively (**Table 3**). Exponential (*R* ^2^> 0.9, *p*< 0.001) and linear (*R* ^2^> 0.9, *p*< 0.01) functions best fit the variation in D_c_ of D0 and of STI, respectively, with irrigation time (**Table 3**).

**Table 3:** Functions describing relations between surface wetted circle diameter and irrigation time.

Treatment	Fitting equations	Time allocation (min)	*R* ^2^	*F*	*P*
D0	*f*(*t*) =65.699−57.694e^-0.011^ *^t^*	t∈(0, 425)	0.951	119.659	<0.001
S1	*f*(*t*) =(0,10) *f*(*t*) =13.348+0.112*t*	t∈(0, 21) t∈(21, 425)	0.919	68.283	<0.01
S2	*f*(*t*) =(0,10) *f*(*t*) =4.943+0.072*t*	t∈(0, 47) t∈(47, 425)	0.979	=186.882	<0.001
S3	*f*(*t*) =(0,10) *f*(*t*) =5.989+0.047*t*	t∈(0, 111) t∈(111, 425)	0.975	76.840	<0.05

D0 is surface drip irrigation (SD), and S1, S2, S3 are STI with sand tube depths of 10 cm, 20 cm, and 30 cm in the in-situ infiltration test.

#### 3.1.2 Surface wetted characteristics after 720 min of irrigation

**Figure 5:**
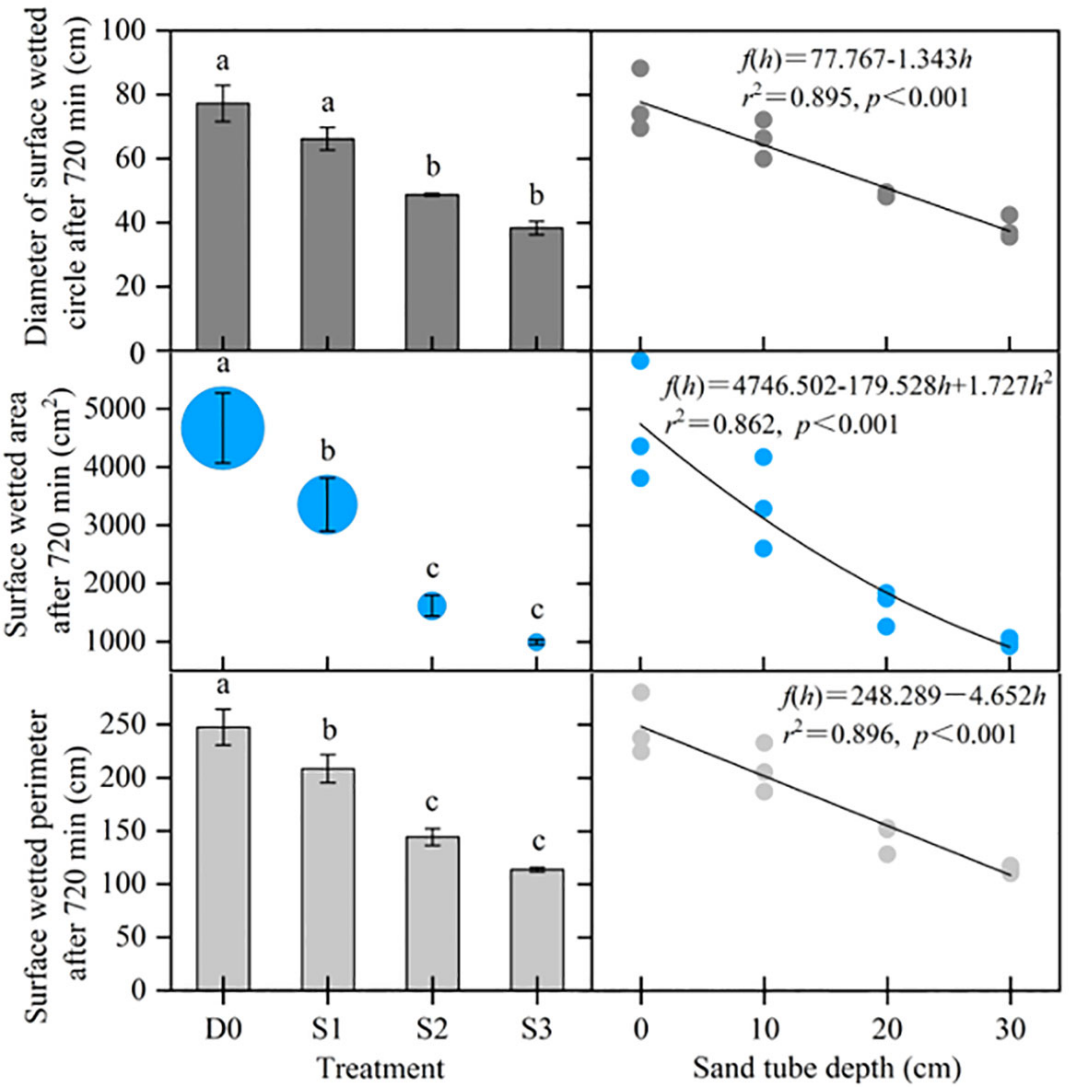
Surface wetted characteristics after 720 min of irrigation. Different lowercase letters indicate significant differences at p< 0.05. Bars indicate the standard error.

Variations in D_c_, wetted area (A_c_), and wetted perimeter (P_c_) of the surface wetted circle in each treatment after 720 min of irrigation are shown in **Figure 5**. With an increase in sand tube depth, D_c_, A_c_, and P_c_ all decreased, and there were significant negative correlations with sand tube depth (*p*< 0.001). In addition, the D_c_ in D0 was not significantly different compared with that in S1 but was significantly different compared with that in S2 and S3, whereas there was no significant difference between S2 and S3. Both A_c_ and P_c_ were significantly greater with SD than with STI. The A_c_ in D0 was 1.4, 2.9, and 4.7 times greater than that in S1, S2, and S3, respectively. Differences in A_c_ between S1 and S2 and S3 were significant but not that between S2 and S3. Differences in P_c_ between S1 and S2 and S3 were significant but not that between S2 and S3.

#### 3.1.3 Characteristics of water infiltration in vertical soil profile at the end of irrigation after 720 min

**Figure 6:**
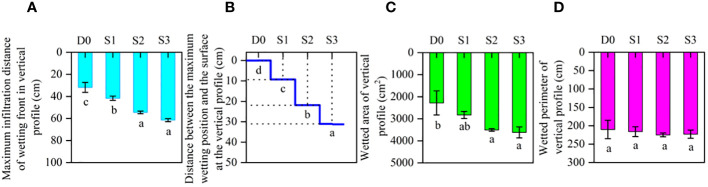
Characteristics of water infiltration in vertical soil profiles. **(A)** Maximum infiltration distance of the wetting front in vertical profile (cm). **(B)** Distance between the maximum wetting position and the surface in vertical profile (cm). **(C)** Wetted area of soil vertical profiles (cm2). **(D)** Wetted perimeter of soil vertical profiles (cm). Different lowercase letters indicate significant differences at p< 0.05. Bars indicate the standard error.

Variations in maximum infiltration distance of the wetting front (D_f_), distance between the maximum wetting location and the surface (D_l_), wetted area (A_p_), and wetted perimeter (P_p_) for soil vertical profiles in each treatment are shown in **Figures 6A–D** The D_f_ increased with increasing of sand tube depth, and compared with SD, STI treatments (S1, S2, and S3) significantly (*p*< 0.05) increased D_f_ by 30.68%, 70.9%, and 93.2%, respectively. The D_f_ was not significantly different between S2 and S3. The D_l_ of treatments changed significantly with the increase in infiltration depth. The maximum infiltration depth of D_l_ was in S3 (31.1 cm), and the minimum was with SD (0 cm). Compared with SD, the A_p_ in S2 and S3 increased significantly by 53.8% and 58.7%, respectively. Compared with D_0_, although differences among treatments were not significant, the P_p_ in S2 and S3 increased by 7.1% and 6.1%, respectively.

### 3.2 Field nitrogen regulation experiments in a jujube tree orchard

#### 3.2.1 SWS

Compared with SD, STI significantly increased (*p<* 0.05) average SWS (Equation 1) over the entire growth period 18.0% in 2021 and 15.6% in 2022 (**Table 4**). The maximum value of SWS in both years was in SN1, and the minimum value was in DN2. Compared with SD, in the leaf emergence period, SWS increased by 22.1% in 2021 and by 12.4% in 2022 in STI treatments. The minimum values of SWS were in SN3 and DN2. In the flowering period, compared with SDI, SWS increased by 19.5% in 2021 and by 16.0% in 2022 in STI treatments. Under the same irrigation pattern, differences in SWS among treatments were not significant, except for SN1 in 2021, which was significantly different compared with that in SN2 and SN3. The lowest SWS of STI treatments was in SN3, which was related to the increased water requirement of jujube trees due to the application of fertilizers during the period. The fruit-swelling period is the longest period with and the highest water demand and thus is the critical period of yield formation for jujube trees. Compared with SD, SWS increased by 14.2% in 2021 and by 20.8% in 2022 (*p<* 0.05) in STI treatments. In the fruit maturation period, compared with SD, SWS in STI treatments increased by 16.1% in 2021 and by 13.3% in 2022. Compared with DN3, SWS in SN3 increased significantly by 11.4% in 2021 and by 14.5% in 2022.

**Table 4:** Effects of different nitrogen treatments on soil water storage (mm) during growth periods of jujube trees under sand tube irrigation and surface drip irrigation in 2021 and 2022.

Treatment	Growth period		Flowering		Fruit swelling		Fruit maturation		Average of entire growth period	
	Leaf emergence									
	2021	2022	2021	2022	2021	2022	2021	2022	2021	2022
SN1	224.0 ab	239.0 a	234.3 a	230.8 a	216.3 a	231.1 a	207.6 a	231.5 a	220.6 a	233.1 a
SN2	212.2 ab	233.7 a	207.7 bc	224.7 ab	197.0 bc	229.0 a	205.4 a	218.1 ab	205.6 b	226.4 ab
SN3	208.4 bc	222.2 ab	214.4 b	214.9 abc	199.4 bc	208.6 b	204.8 a	218.6 ab	206.7 b	216.1 b
SN4	231.3 a	234.8 a	217.1 ab	238.0 a	203.9 ab	226.4 ab	210.9 a	229.3 a	215.8 ab	232.1 a
DN1	182.9 d	220.8 ab	190.5 cd	182.5 d	168.1 e	187.3 c	171.9 b	205.8 bcd	178.4 c	199.1 c
DN2	162.4 e	187.9 c	174.1 d	203.5 bcd	175.4 de	182.6 c	179.4 b	187.3 d	172.8 c	190.3 c
DN3	180.8 de	207.8 b	192.0 cd	195.4 cd	186.7 cd	187.3 c	182.2 b	191.0 cd	185.4 c	195.4 c
DN4	191.3 cd	210.5 b	174.3 d	201.5 bcd	185.1 cd	183.8 c	180.0 b	208.1 bc	182.7 c	200.9 c

Different lowercase letters indicate significant differences at p< 0.05 in the same column.

#### 3.2.2 Leaf chlorophyll content and NBI

**Figure 7:**
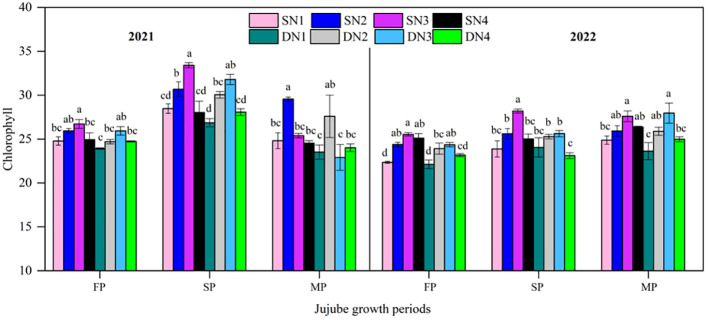
Leaf chlorophyll content in different nitrogen treatments with two irrigation methods during jujube growth periods in 2021 and 2022. FP, flowering period; SP, fruit-swelling period; MP, fruit maturation period. Different lowercase letters indicate significant differences at *p*< 0.05. Bars indicate the standard error. The same as below.

As jujube tree growth progressed, leaf chlorophyll content of treatments were showed an increase and then decline in 2021 and increased and then stabilized in 2022 (**Figure 7**). Compared with SD, STI increased leaf chlorophyll content over the entire growth period by 4.2% in 2021 and by 3.7% in 2022. In 2021, compared with SD, STI increased chlorophyll content by 3.1% at flowering, 3.3% at fruit swelling, and 6.4% at maturation. Similarly, in 2022, STI increased leaf chlorophyll content by 4.7% at flowering, 2.3% at fruit swelling, and 3.7% at maturation. Leaf chlorophyll content increased and then decreased with increasing N application with both STI and SD in 2021 and 2022. The maximum leaf chlorophyll content in 2021 and 2022 at flowering and fruit-swelling periods was in SN3. At fruit maturation, maximum values of chlorophyll content were in SN2 and DN3 in both years. In addition, leaf chlorophyll content in SN3 was significantly different compared with that in SN1 at both flowering and fruit swelling. At fruit maturation, leaf chlorophyll content in SN3 was significantly different from that in SN1 only in 2022. Minimum leaf chlorophyll content was in N1 treatments in both years in all growth periods under both irrigation patterns, except for the minimum value in DN3 at fruit maturation in 2021.

**Figure 8:**
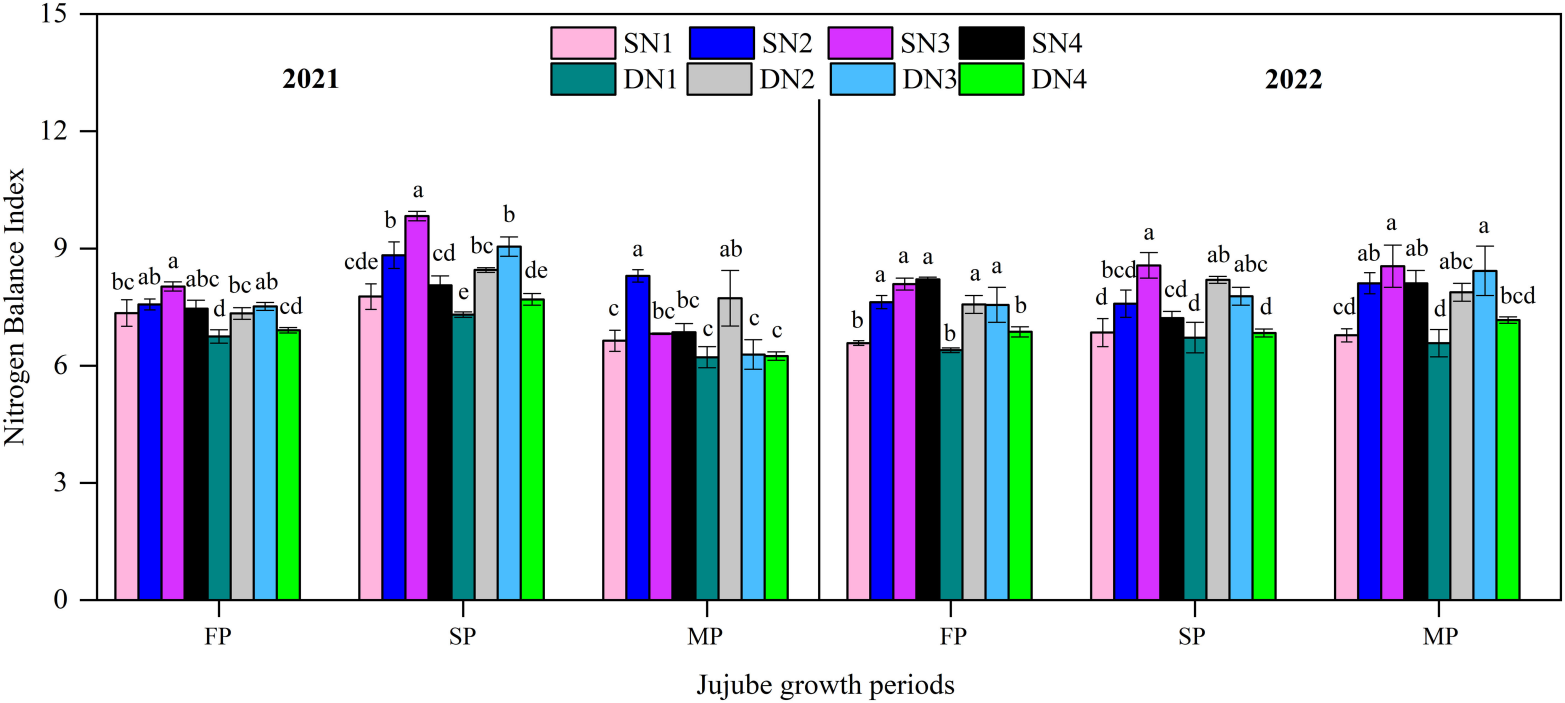
Nitrogen balance index in different nitrogen treatments with two irrigation methods during jujube growth periods in 2021 and 2022. Different lowercase letters indicate significant differences at p< 0.05. Bars indicate the standard error.

**Figure 8** showed the changes of NBI at different growth periods for each treatment over the two years. For NBI, STI increased 6.9% (2021) and 4.9% (2022) on average compared with SD over the entire growth periods. In addition, NBI showed an increasing and then decreasing trend with increasing nitrogen application in this two irrigation methods. In both years, the SN3 was significantly higher than the SN1 at flowering and fruit swelling. Although SN4 applied more N than SN3, the former was significantly higher than the latter at fruit swelling. For SD, DN2 was the largest in 2021 and DN3 was the largest in 2022 during flowering and fruit swelling, and both were significantly higher than DN1 and DN4.

#### 3.2.3 Net photosynthesis rate

**Figure 9:**
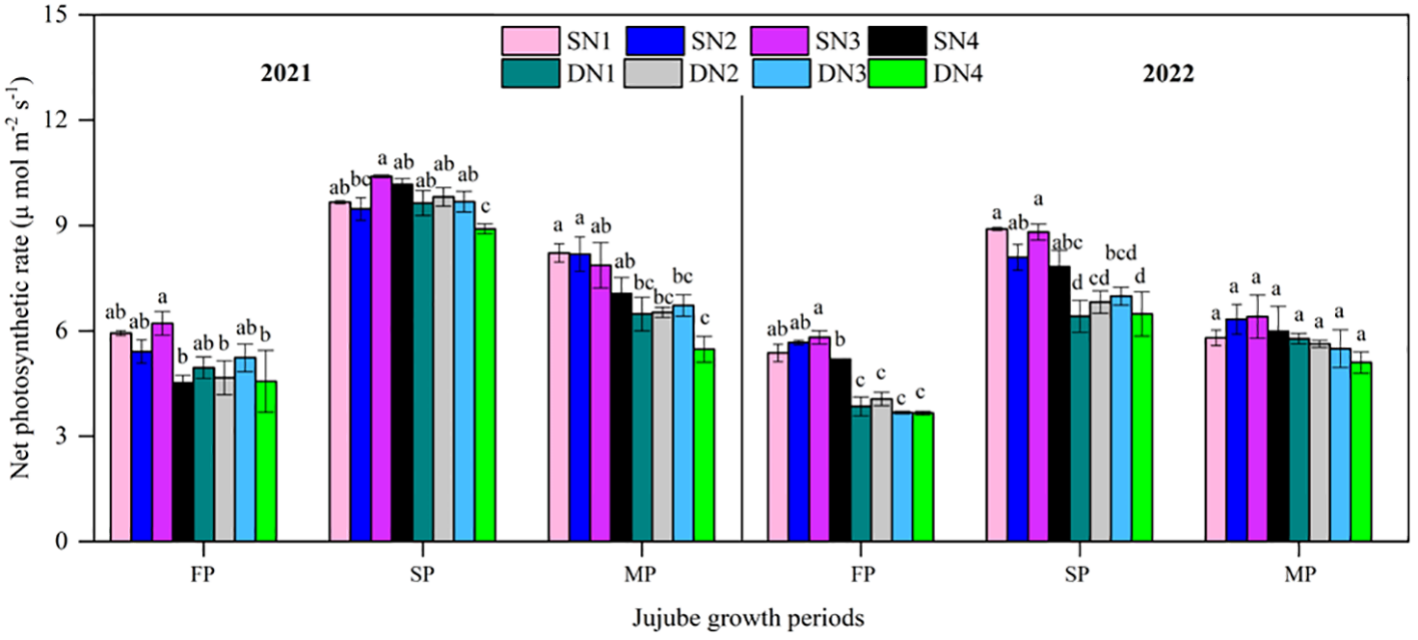
Net photosynthetic rate of different nitrogen treatments with two irrigation methods during jujube growth periods in 2021 and 2022. Different lowercase letters indicate significant differences at p< 0.05. Bars indicate the standard error.

The net photosynthesis rate (Pn) in each treatment increased and then decreased during jujube growth in 2021 and 2022 (**Figure 9**). The maximum Pn was in the fruit swelling period, with 8.9–10.4 (2021) and 6.4–8.9 μmol m^-2^ s^-1^ (2022), and the minimum Pn was in the flowering period, with 4.5–6.2 (2021) and 3.7–5.8 μmol m^-2^ s^-1^ (2022). In 2021, compared with SD, Pn increased by 13.8% at flowering,4.4% at fruit swelling, and 24.3% at fruit maturation with STI. In 2022, compared with SD, Pn increased by 44.7% at flowering, 26.0% at fruit swelling, and 11.5% at fruit maturation. In 2021, the Pn between SN3 and SN4 at flowering and between SN2 and SN3 at fruit swelling was significantly different, whereas differences between other treatments were not significant. In 2022, the Pn in SN3 and SN4 at flowering was significantly different, whereas differences between other treatments were not significant. There were no significant differences in Pn among SD treatments for the entire growth period in either year.

#### 3.2.4 Jujube yield, crop actual evapotranspiration, WUE, and NUE

Jujube yield (Y), crop actual evapotranspiration (ET_a_, Equation 2), WUE (Equation 3), IWUE (Equation 4) and NUE (Equation 5) of each treatment are shown in **Table 5**. Yield increased and then decreased with increasing N amount for the same irrigation method. The yield in STI increased by 39.1% in 2021 and by 36.5% in 2022 compared with that in SD. In 2021, yield in SN3 was significantly higher than that in other treatments. In 2022, yield in SN3 was not significantly different compared with that in SN2 but was significantly different compared with that in the other treatments. Among SD treatments, the highest yields were in DN3 in both years, although differences among treatments were not significant. Compared with SD, ET_a_ in STI decreased by 3.4% in 2021 and by 2.1% in 2022. In 2021, ET_a_ in SN3 increased by 0.6% compared with that in DN3, but in 2022, ET_a_ in SN3 decreased by 4.8% compared with that in DN3, although the difference was not significant. Compared with SD, WUE in STI treatments increased by 44.3% in 2021 and by 39.7% in 2022. In addition, with both STI and SD, WUE increased and then decreased with increasing N application in 2021 and 2022. In both years, maximum WUE values were in SN3, and minimum WUE values were in DN1. In both years, WUE in STI treatments (except for SN1 and SN4 in 2021 and SN1 in 2022) was significantly higher than that in SD treatments. The IWUE is determined by the ratio of yield to irrigation amount. With both irrigation methods, IWUE increased and then decreased, with the largest value in SN3 and the smallest in DN1. In addition, IWUE with STI was 1.4 times higher than that with SD in both years. The NUE with STI was 2.5 times (2021) and 1.6 times (2022) higher than that with SD. The maximum NUE value was in SN3, and the minimum NUE value was in DN4 in both years. The NUE values in STI treatments increased and then decreased, whereas NUE values with SD showed instability (maximum values were in DN2 in 2021 and in DN3 in 2022).

**Table 5:** Effects of different nitrogen treatments on yield, crop actual evapotranspiration (ET_a_), water use efficiency (WUE), and nitrogen use efficiency (NUE) of jujube trees under two irrigation methods in 2021 and 2022.

Treatment	2021	ET_a_ (mm)	WUE (kg m^-3^)	IWUE (kg m^-3^)	NUE (kg kg^-1^)	2022	ET_a_ (mm)	WUE (kg m^-3^)	IWUE (kg m^-3^)	NUE (kg kg^-1^)
	Y (kg ha^-1^)					Y (kg ha^-1^)				
SN1	8,782 cd	438.9 abc	2.0 cd	2.4 cd	–	9,387 bcd	424.4 bcd	2.2 bcd	2.6 bcd	–
SN2	10,325 b	388.3 c	2.7 ab	2.9 b	5.4 ab	11,560 ab	440.5 abc	2.6 ab	3.2 ab	7.6 ab
SN3	12,497 a	404.6 abc	3.1 a	3.5 a	9.8 ab	13,149 a	447.0 ab	2.9 a	3.7 a	9.9 a
SN4	9,677 bc	426.6 abc	2.3 bc	2.7 bc	1.9 b	10,177 bc	408.0 cd	2.5 abc	2.8 bc	1.7 b
DN1	6,801 e	444.1 ab	1.5 d	1.9 e	–	7,008 e	404.3 d	1.7 d	1.9 e	–
DN2	7,677 de	412.4 abc	1.9 cd	2.1 de	3.1 b	8,360 cde	455.9 ab	1.8 d	2.3 cde	4.7 ab
DN3	7,906 de	402.0 bc	2.0 cd	2.2 de	2.9 b	9,260 cde	469.3 a	2.0 d	2.6 cde	5.9 b
DN4	7,163 e	457.5 a	1.6 d	2.0 e	0.8 b	7,649 de	427.8 bcd	1.8 d	2.1 de	1.3 b

Different lowercase letters indicate significant differences at p< 0.05.

Additionally, compared with N0, yield and WUE increased and then decreased with increasing N amount with the two irrigation methods. Increases in both yield and WUE were higher with STI than with SD. Specifically, the increases in yield were 23.4% in 2021 and 23.9% in 2022 with STI and 11.6% in 2021 and 20.2% in 2022 with SD. The increases in WUE were 33.6% in 2021 and 22.1% in 2022 with STI and 19.0% in 2021 and 7.7% in 2022 with SD. With STI, the maximum was in SN3, and the minimum was in SN4, and with SD, the maximum was in DN3, and the minimum was in DN4. When N treatments were compared with the previous level of treatment, yield and WUE increased in N2 and N3 treatments (SN2, SN3, DN2, and DN3) but decreased in N4 treatments (SN4 and DN4). With STI and SD at the same N level, the increases in yield were 29.2% to 57.9% in 2021 and 33.1% to 42.0% in 2022. The increases in WUE were 30.8% to 52.3% in 2021 and 27.1% to 49.0% in 2022. In addition, the maximum increases in yield and WUE were in SN3.

#### 3.2.5 Pearson’s correlation analyses

**Figure 10:**
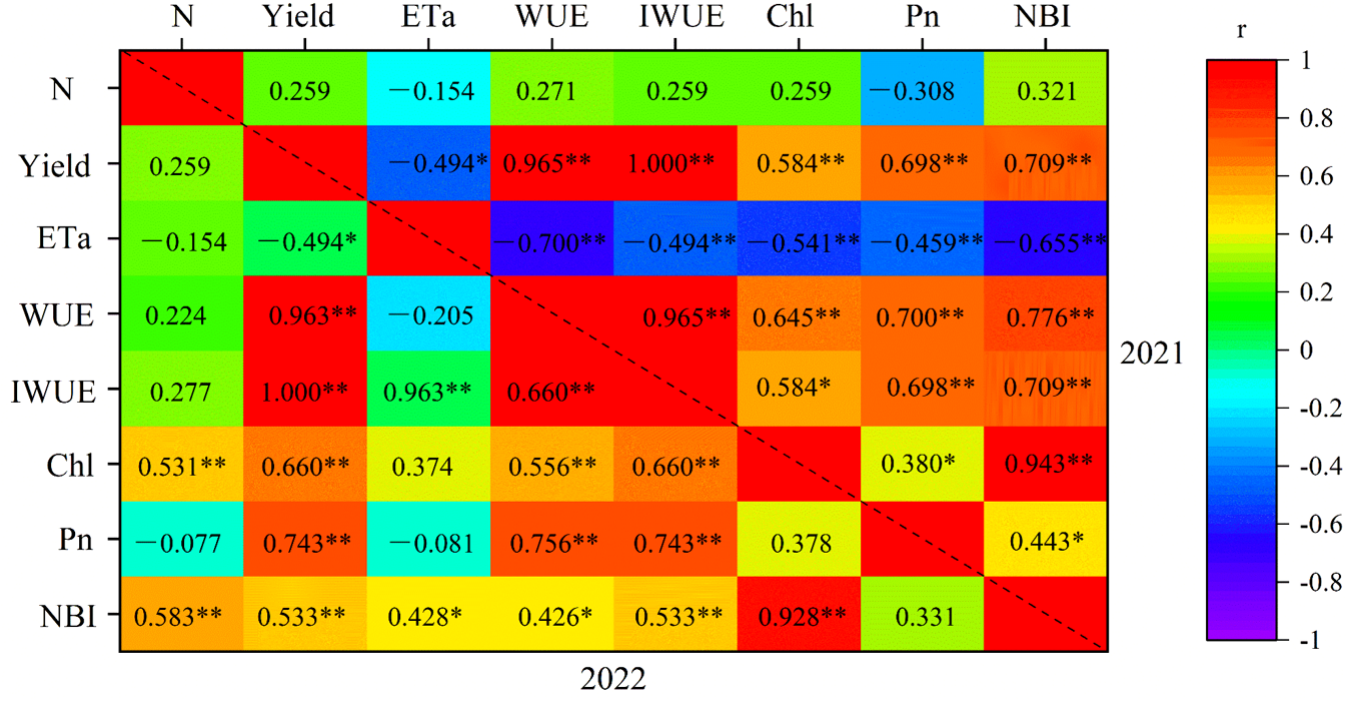
Pearson correlations (correlation coefficient *r*) between indicators under different treatments. *p< 0.05; **p< 0.01. N, nitrogen amount; Yield, jujube yield; ET_a_, crop actual evapotranspiration; WUE, water use efficiency; IWUE, irrigation water use efficiency; Chl, Leaf chlorophyll content; Pn, net photosynthetic rate.

Pearson correlations between indicators under different treatments are shown in **Figure 10**. Yield was highly significantly (*p*< 0.001) positively correlated with WUE, IWUE, Chl, NBI, and Pn in both years. In 2021, ET_a_ was highly significantly negatively correlated with WUE and Chl and significantly negatively correlated with IWUE and Pn, but not in 2022. In addition, WUE was highly significantly positively correlated with IWUE, Chl, and Pn in both years. Simultaneously, IWUE was highly significantly positively correlated with both Chl, and Pn. However, ET_a_ and yield were not correlated.

#### 3.2.6 Comprehensive analysis of economic benefits under two irrigation methods

There are larger differences in irrigation methods between STI and SD. The main difference between the two irrigation methods is mainly the different distribution of irrigation water in the soil. This ultimately leads to an increase in soil water storage for STI compared with SD. Additionally, there will be differences in total inputs between the two irrigation methods. The pursuit of net profit is the primary interest of the farmers. As shown in **Table 6**, the labor and consumables (fine sand) for arranging the sand tube increase their investment by $208 in 2021 compared with SD. In fact, fine sand is more abundant in the study area, which can reduce the cost of consumables. In 2022, the total investment was the same for both irrigation methods under the same fertilizer conditions. STI increased the net income by 23.5% (2021) and 20.3% (2022) compared with SD, respectively. The main reason for this is that the yield in STI increased by 39.1% in 2021 and by 36.5% in 2022 compared with that in SD (**Table 5**). The input-output ratio was also an important indicator of economic benefits. In both years, the input-output ratio of STI was lower than that of SD. When analyzed comprehensively, the SN3 treatment had the highest net income and the lowest input-output ratio in both years.

**Table 6:** Analysis of economic benefits (Equation 6) of sand tube irrigation and surface drip irrigation for jujube in 2021 and 2022.

Year	Treatment	Input ($ ha^−1^)				Output ($ ha^−1^)		Net income	Input-output ratio
		Water cost	Nitrogen cost	Labor and consumables	Total investment	Price ($ kg^−1^)	Total income	($ ha^−1^)	
2021	SN1	171	0	2,292	2,463	1	8,132	5,669	1∶3.30
	SN2	171	181	2,292	2,644	1	9,561	6,917	1∶3.62
	SN3	171	302	2,292	2,765	1	11,572	8,807	1∶4.19
	SN4	171	363	2,292	2,825	1	8,961	6,136	1∶3.17
	DN1	171	0	2,084	2,254	1	6,298	4,043	1∶2.79
	DN2	171	181	2,084	2,436	1	7,109	4,673	1∶2.92
	DN3	171	302	2,084	2,557	1	7,321	4,764	1∶2.86
	DN4	171	363	2,084	2,617	1	6,633	4,016	1∶2.53
2022	SN1	171	0	1,875	2,046	1	8,692	6,646	1∶4.25
	SN2	171	181	1,875	2,227	1	10,704	8,477	1∶4.81
	SN3	171	302	1,875	2,348	1	12,176	9,828	1∶5.19
	SN4	171	363	1,875	2,409	1	9,424	7,016	1∶3.91
	DN1	171	0	1,875	2,046	1	6,489	4,443	1∶3.17
	DN2	171	181	1,875	2,227	1	7,741	5,514	1∶3.48
	DN3	171	302	1,875	2,348	1	8,574	6,226	1∶3.65
	DN4	171	363	1,875	2,409	1	7,083	4,674	1∶2.94

## 4 Discussion

### 4.1 STI reduces surface wetted area and increases infiltration capacity

In this study, an *in-situ* test was conducted to optimize STI parameters, and then, infiltration in surface soil and subsurface soil vertical profiles was investigated in different treatments. Soil infiltration with STI has been investigated in the laboratory (Meshkat et al., 1998; Meshkat et al., 1999; Meshkat et al., 2000). However, in this study, the *in-situ* infiltration experiment was conducted in the field, with results reflecting actual soil structure and infiltration processes. The field investigation has important practical significance for selecting optimal sand tube parameters for field cultivation of jujube trees. In this study, STI significantly reduced A_c_ and increased maximum infiltration distance of the wetting front (**Figures 5**, **6**). The results are in line with those of Bai et al. (2022), who reported that STI reduces A_c_ by 50.6% compared with SD. According to Meshkat et al. (2000), STI reduces evaporation by 39.8%, similar to the findings of the present study. Wang et al. (2020) found that introduction of infiltration holes significantly increased soil moisture (0 to 100 cm) in a semiarid, sloped shrubland. Those results are consistent with STI significantly increasing SWS compared with that with SD in this study.

The STI depth of 20 cm was selected for the cultivation of jujube trees in the field for the following reasons: (1) the main root layer of jujube trees is at 0–60 cm (Wang et al., 2015), (2) the root distribution of jujube trees and the formation of underground pore space may result in preferential flow that leads to deep seepage (Benegas et al., 2014; Cui et al., 2021; Li et al., 2023; Luo et al., 2023), (3) there was no significant difference in surface wetted area, maximum infiltration distance of the wetting front, and wetted area for vertical profiles between S2 and S3 in the *in-situ* test (**Figures 5**, **6**), and (4) to reduce the investment in manpower and materials. In a similar study, Sun et al. (2016) developed an indirect subsurface drip irrigation system with vertical tubes (sand filled at the bottom) set at a depth of 25 cm. In addition, Wang et al. (2021) developed vertical-tube irrigation in jujube trees with a vertical-tube depth of 40 cm. The variation in technical parameters may be related to soil type, drip emitter flow rate, meteorological conditions, and tree age, among other factors, and which also needs to be determined on a case-by-case basis in conjunction with soil infiltration tests.

### 4.2 Nitrogen regulation with STI increases jujube chlorophyll content, NBI, and net photosynthetic rate

Because N is an essential component of chlorophyll molecular structure, N deficiency usually results in stunted plant growth and chlorotic leaves due to poor assimilate formation, which ultimately leads to premature flowering and a shortened growth cycle (Larimi et al., 2014). In this study, chlorophyll content was highly significantly (*p*< 0.001) correlated with yield in 2021 and 2022 (**Figure 10**), which is in line with the findings on the application of nitrogen to jujube tree (Dai et al., 2019; Zhang et al., 2023).

In this study, chlorophyll content increased with increasing N application, but the highest N amount did not result in further increase in chlorophyll content. However, Pramanik and Bera (2013) found the highest N application had the highest chlorophyll content, although not the highest yield. The light energy converted into chemical energy by photosynthesis is absorbed by the plant green pigment chlorophyll (Simkin et al., 2022). In this study, appropriate N application was beneficial and increased chlorophyll content (**Figure 7**). NBI is an important indicator for assessing crop nitrogen deficiency and nitrogen management (Fan et al., 2022). Sun et al. (2018) showed that nitrogen sufficiency and deficiency were closely related to leaf color and chlorophyll content. In the present study, chlorophyll content was highly significantly positively correlated with NBI (**Figure 10**), and increasing nitrogen significantly increased chlorophyll content and NBI. In addition, the present study was also found that excessive nitrogen application decreased leaf chlorophyll content and NBI, which was similar to the findings of Bo et al. (2021). Leaf net photosynthetic rate varied inconsistently under both irrigation methods (**Figure 9**). During the flowering stage, Pn increased and then decreased with increasing N application. However, Pn decreased with increasing N application at maturity. The main reason for this result was because the low-N application had low fruit set percentage and less dry matter accumulation, which in turn reduced the excessive consumption of soil nutrients, and which was an important reason for the higher chlorophyll content under low N conditions (**Figure 7**).

### 4.3 Nitrogen regulation with STI increases jujube yield, WUE, and NUE

STI reduced the average ET_a_ (**Table 5**), which was associated with reduced evaporation of surface soil moisture. Evaporation from shallow soils increases due to the exposure of surface soils to solar radiation and soil capillary action (Andersen, 1968; Islam and Morimoto, 2015). These processes further increase soil moisture dissipation, leading to soil moisture deficit, and are one of the important factors associated with yield reduction under SD. In this study, there was no significant difference in ET_a_ between SN3 and DN3 (**Table 6**), but according to the *in-situ* infiltration test, the A_c_ of STI was significantly smaller than that of SD (**Figure 5**). This result also indicated that surface evaporation was greater in DN3 than in SN3, which was an important reason why the yield and WUE in SN3 were significantly higher than those with SD (**Table 5**). In this study, the average WUE with STI was 1.4 times greater than that with SD. This result is consistent with that of Wang et al. (2021c) who found that WUE of vertical-tube irrigation is 1.4–4.3 times that of SD. For average SWS, the maximum value in both years was in SN1, which was significantly higher than that in SN3. This result was related to the increase in N application, which promoted jujube growth and increased water consumption under the same irrigation condition (**Table 5**).

In related studies, crop yields do not increase linearly with increasing N applications (Széles et al., 2012; Guo et al., 2021). In the present study, jujube fruit yield increased and then decreased slightly with increasing N application. Similarly, Lu et al. (2021) showed that grain yields tended to increase and then remain stable with increasing N amount. In this study, WUE in SN3 increased significantly compared with that in SN1, whereas WUE in SN4 was not significantly different compared with that in SN1. Therefore, suitable N application significantly increased WUE but excessive N decreased WUE. The IWUE with STI was 1.4 times higher than that with SD in both years (**Table 5**), which indicated that STI is an advanced irrigation technology that increases water productivity compared with that with SD. The NUE with STI was 2.2 times higher than that with SD, which indicated that STI effectively improves NUE. The increases in SWS and yield with STI were important reasons for the increase in NUE (**Table 4**, **Table 5**). In this study, NUE increased and then decreased with increasing N application under both irrigation patterns, which are results similar to those reported by Ma et al. (2022).

In conclusion, STI combined with appropriate N increased jujube yield, WUE and NUE compared with SD. Therefore, STI can be used as an alternative technology to SD in jujube cultivation. However, limitations of this study are that the technical parameters of the sand tube are affected by soil type, physical properties, and crop species and that the nitrogen amounts applied are affected by soil fertility, crop species, and local fertilization experience.

## 5 Conclusion

In this study, field *in-situ* soil water infiltration tests were first conducted to optimize sand tube technical parameters. Then, nitrogen regulation experiments with sand tube irrigation (STI) and surface drip irrigation (SD) were conducted in the field using the preferred sand tube parameters. In the field *in-situ* infiltration tests, STI significantly reduced soil surface wetted area and increased water infiltration depth, compared with SD. The optimal sand tube depth for cultivating jujube trees was 20 cm. In field-cultivated jujube, STI was more effective than SD in increasing SWS, chlorophyll content, and net photosynthetic rate. STI also increased jujube fruit yield, water and nitrogen use efficiencies, and net income. Increasing nitrogen application increased chlorophyll content, nitrogen balance index, net photosynthetic rate, jujube fruit yield, and water and nitrogen use efficiencies, but excessive N application caused an unstable decline in each index under both irrigation patterns. On the basis of a comprehensive analysis, STI combined with N3 had the highest yield, WUE, NUE, and net income. This study provides a new alternate irrigation method for jujube trees that can replace SD. And, through nitrogen regulation experiments, STI combined with N3 is the optimal irrigation and nitrogen management strategy for jujube trees in this region. However, the shortcoming of this study is that there is a deviation between hole application of fertilizer and conventional water and fertilizer integration (fertilizer dissolved in water for irrigation). This may affect the nitrogen use efficiency. In the future, it is recommended to use sand tube irrigation technology in combination with water and fertilizer integration technology in large scale application.

## Data availability statement

The raw data supporting the conclusions of this article will be made available by the authors, without undue reservation.

## Author contributions

YB: Conceptualization, Data curation, Formal analysis, Funding acquisition, Methodology, Writing – original draft, Writing – review & editing. HZ: Conceptualization, Methodology, Supervision, Writing – original draft, Writing – review & editing. SJ: Conceptualization, Funding acquisition, Methodology, Supervision, Writing – original draft. DS: Formal analysis, Writing – review & editing. JZ: Formal analysis, Writing – review & editing. XZ: Data curation, Writing – review & editing. XF: Data curation, Funding acquisition, Writing – original draft. XW: Data curation, Writing – review & editing. CX: Data curation, Writing – review & editing. RC: Data curation, Writing – review & editing.
